# Cell-Free bone regeneration using dental MSCs secretomes from pulp and gingiva in rabbit tibial defects

**DOI:** 10.1038/s41598-025-15924-7

**Published:** 2025-08-28

**Authors:** Dina Kamal, Dina Rady, Sara El Moshy, Israa Ahmed Radwan, Nermeen El-Moataz Bellah Ahmed, Al-Hassan Soliman Wadan, Samah S. Mehanny, Lobna Salah Eldin

**Affiliations:** 1 Oral Biology Department, Faculty of Dentistry, Cairo University, Cairo, Egypt; 2 Stem cells and Tissue Engineering Research Group, Faculty of Dentistry, Cairo University, Cairo, Egypt; 3 Oral Biology Department, Faculty of Dentistry, German University in Cairo, Cairo, Egypt; 4 Oro-dental Genetics Department, Human Genetics and Genome Research Institute, National Research Centre, Cairo, Egypt; 5 Molecular biology research laboratory, Faculty of Oral and Dental Medicine, Misr International University, Cairo, Egypt; 6 Oral Biology Department, Faculty of Dentistry, Galala University, Galala City, Suez Governorate Egypt

**Keywords:** Mesenchymal stem cell secretome, Bone regeneration, Cell free-therapy, Mineral apposition rate, Osteogenic markers., Adult stem cells, Stem-cell niche

## Abstract

Mesenchymal stem cells (MSCs)-secretome represent a promising cell-free strategy for bone regeneration, overcoming cell therapies’ drawbacks. This study compares the bone repair capabilities of secretomes derived from human dental pulp stem cells (hDPSCs) and human gingival stem cells (hGMSCs) in a rabbit tibial defect model. Secretomes were prepared, and levels of RUNX, osterix, and alkaline phosphatase (ALP) levels were quantified using ELISA. Six-mm defects were created in rabbit tibiae treated with either hDPSCs or hGMSCs secretomes, collagen scaffolds, or left untreated. The mineral apposition rate (MAR) was assessed using fluorescent labeling. Histomorphometric analysis (including bone area percentage, mature/immature bone and bone marrow quantification) and qRT-PCR for osteocalcin were conducted at 3 and 6 weeks. Results showed significantly higher concentrations of RUNX, osterix, and ALP in hDPSCs secretome compared to hGMSCs. Additionally, defects treated with hDPSCs secretome exhibited a higher MAR and greater new bone formation, accelerated maturation (higher mature bone area; *p* < 0.05), and reduced bone marrow spaces at 3 weeks than those treated with hGMSCs, collagen, or control groups. By 6 weeks, both secretomes achieved comparable bone maturation (83–85% bone area), significantly surpassing controls. Both secretomes upregulated osteocalcin gene expression. These findings show the therapeutic potential of dental MSCs secretomes, and particularly hDPSCs secretomes, as a cell-free, clinically relevant method for improving bone regeneration. This approach addresses limitations associated with traditional bone grafting and possibly presents a new pathway for regenerative therapies in dentistry and orthopedics.

## Background

Bone defects result from different causes, such as infections, trauma, congenital malformations, tumor resection, and reconstructive surgery. Bone grafting is the most used surgical method to reinforce bone healing^1^. Worldwide, more than two million bone grafting procedures are performed annually^2^. Different types of bone grafts are available, but each has its drawbacks, such as donor site morbidity, limited bone amount, and post-operative complications for autogenous bone grafts. The limitations of allografts and xenografts include the possibility of pathogen transfer and host immune rejection risk^3^. Therefore, bone regeneration has become a significant goal to provide an effective alternative approach to promote bone healing^4^.

Mesenchymal stem cells (MSCs), particularly those derived from dental tissues, have shown remarkable potential in bone regeneration^5^. Dental MSCs, including dental pulp stem cells (DPSCs) and gingival-derived MSCs (GMSCs), are distinguished by their capacity to restore various mesenchymal tissues and are considered a safe and non-invasive source of human stem cells^6–8^. Although bone marrow-derived MSCs (BM-MSCs) are the most studied, dental MSCs present a promising future due to their easier isolation and successful applications in regenerative medicine^9,10^.

**Fig. 1:**
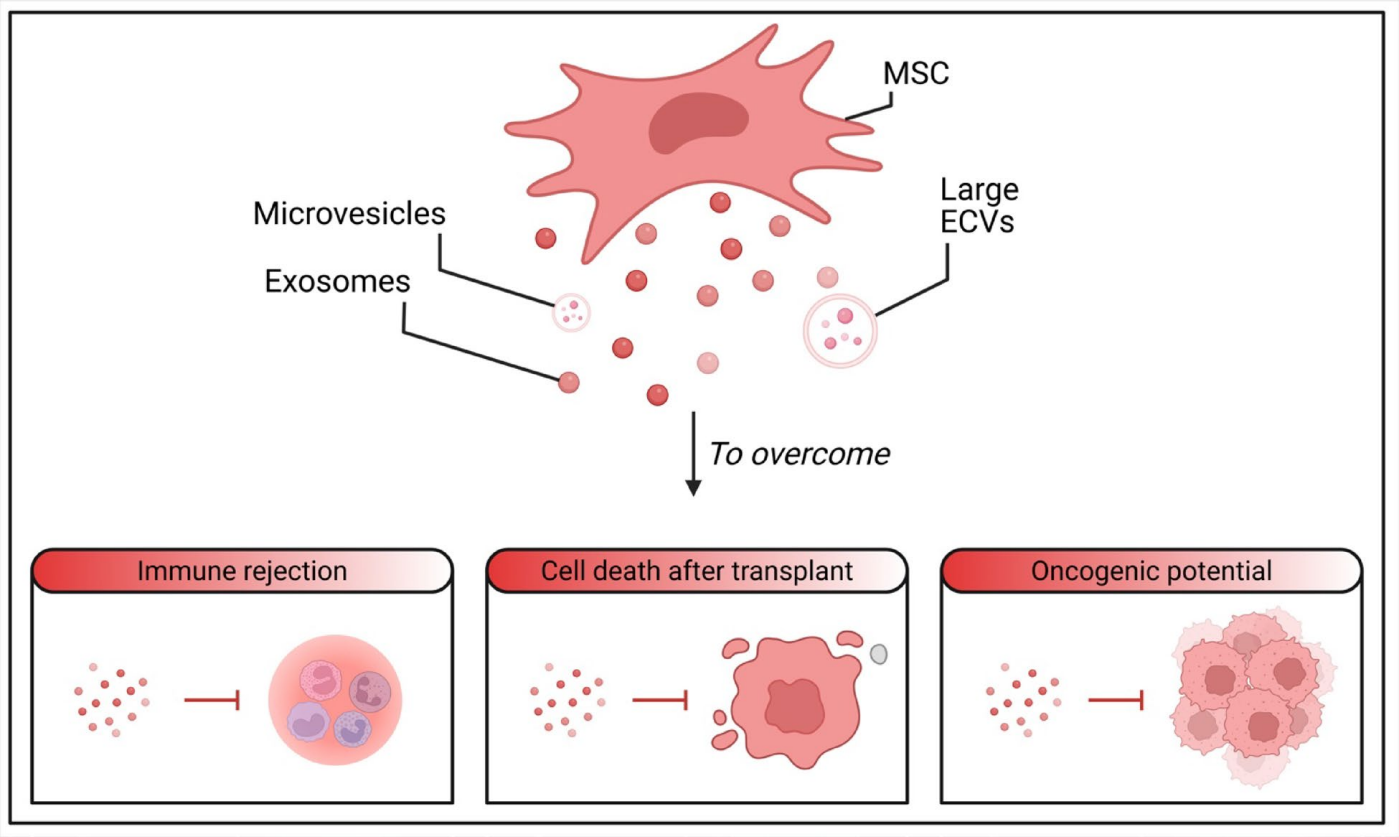
A diagram showing cell-free regenerative medicine approaches utilizing the secretome from stem cells overcome the limitations of cell-based therapies.

The primary impact of MSCs is mediated via paracrine processes, releasing various factors rather than direct differentiation. These factors can induce tissue repair even without stem cells^11^. The secretome of MSCs, which includes exosomes, large extracellular vesicles, and microvesicles, has gained significant attention as an alternative treatment modality^12^. The secretome can be produced at higher rates and shows less immunogenicity due to lower expression of cell surface proteins^11^. Cell-free regenerative medicine approaches utilizing the secretome from stem cells overcome the limitations of cell-based therapies, such as the oncogenic potential of cultured cells, immune rejection, and stem cell death after transplantation^13–15^ (Fig. 1). Therefore, this study seeks to evaluate and compare the bone regenerative potential of secretomes derived from human dental pulp stem cells (hDPSCs) and human gingival mesenchymal stem cells (hGMSCs) in a rabbit tibial bone defect model. By investigating these secretomes, we aim to identify novel therapeutic strategies for enhancing bone repair and regeneration, addressing a critical need in orthopedic and dental medicine. Our findings may pave the way for innovative, cell-free approaches in regenerative medicine, offering significant clinical implications for treating bone defects.

## Methods

### Secretomes preparation

Both DPSCs and GMSCs secretomes were sourced from human donors. All methods involving human donors were carried out in accordance with relevant guidelines and regulations. All experimental protocols were done at the Stem Cell Lab, National Research Centre. Informed consent was obtained from all donors after receiving full information about the experimental purpose of the donation, potential research applications. Stem cells starvation for 24 h was done upon reaching 60% confluency at the 5th passage by shifting to serum-free DMEM. The medium was concentrated about 40-fold by using amicon Ultra-15 centrifugal filter unit with an ultracel-3 membrane (Millipore, Billerica, MA) for medium collection and concentration. The addition of 10 L/mL of Halt protease inhibitor cocktail (Thermo Scientific, USA) to the collected secretome was done, followed by the usage of Coomassie (Bradford) protein assay kit (Thermo Scientific, USA) to determine the protein concentration. The final protein concentration was adjusted to 3 µg/ml^16^.

### Enzyme-linked immunosorbent assay (ELISA)

The expression of alkaline phosphatase (ALP), osterix, and RUNX in hDPSCs and hGMSCs secretome was detected using an ELISA kit (Takara). Standard protein samples were diluted 1:50 with dilution buffer, and a standard curve was drawn. Test samples were diluted at 1:100 with PBS (pH 7.2), and 100 µl of the solution was added to each well. After incubation with the test solution and TMB chromogenic substrate, absorbance values were measured at 495 nm, and protein concentration was calculated according to the standard curve^17^.

### Experimental animals

Healthy male New Zealand white rabbits weighing about 2.5–3.5 kg, which typically corresponds to an age of approximately 3–6 months were used. The present work was conducted in accordance with the regulations and guidelines of the Institutional Animal Care and Use Committee at Cairo University. The Institutional Animal Care and Use Committee (IACUC)-Cairo University approved the study procedures with numbers (GU-III-F-C-62-24). The ARRIVE guidelines have been followed in this animal study.

### Sample size calculation

To evaluate the bone regenerative potential of either hDPSCs secretome or hGMSCs secretome in rabbits’ tibial bone defects, a sample size of 6 defects in each group was deemed adequate. This sample size was sufficient to detect an effect size of 0.82 with 80% power and an α error probability of 0.05. The effect size was calculated from the mean and SD from a previous study^18^. For the MAR, 12 rabbits each rabbit providing 2 defects (one in each tibia) were used (with 24 defects divided among four groups). For histomorphometric analysis of the area percentage of newly formed bone and qRT-PCR analysis, seven defects for each group in each time duration were sufficient to detect an effect size of 0.74 with 80% power and an α error probability of 0.05. The effect size was calculated from the mean and SD from a previous study^19^. Therefore, 28 rabbits each rabbit providing 2 defects (one in each tibia) were used (with 56 defects divided among four groups). Half of the rabbits were euthanized after three weeks, and the other half after six weeks. G*Power software was used to determine the proper sample size.

### Tibial bone defects induction and animal grouping

Surgeries were performed at the animal house of the Faculty of Medicine, Cairo University. Rabbits were anesthetized using ketamine chlorhydrate (0.08 ml/100 gm body weight) combined with xylazine 2% (0.04 ml/100 gm body weight) via the intramuscular route. After disinfection and shaving, a 2–3 cm incision was made to expose the tibiae. A defect of six mm diameter on each tibia at its medial surface was created using a trephine bur (Meisinger, Germany) attached to a low-speed handpiece connected to a 2000-rpm micro-motor. Continuous irrigation with saline solution prevented overheating^20^.

**Fig. 2:**
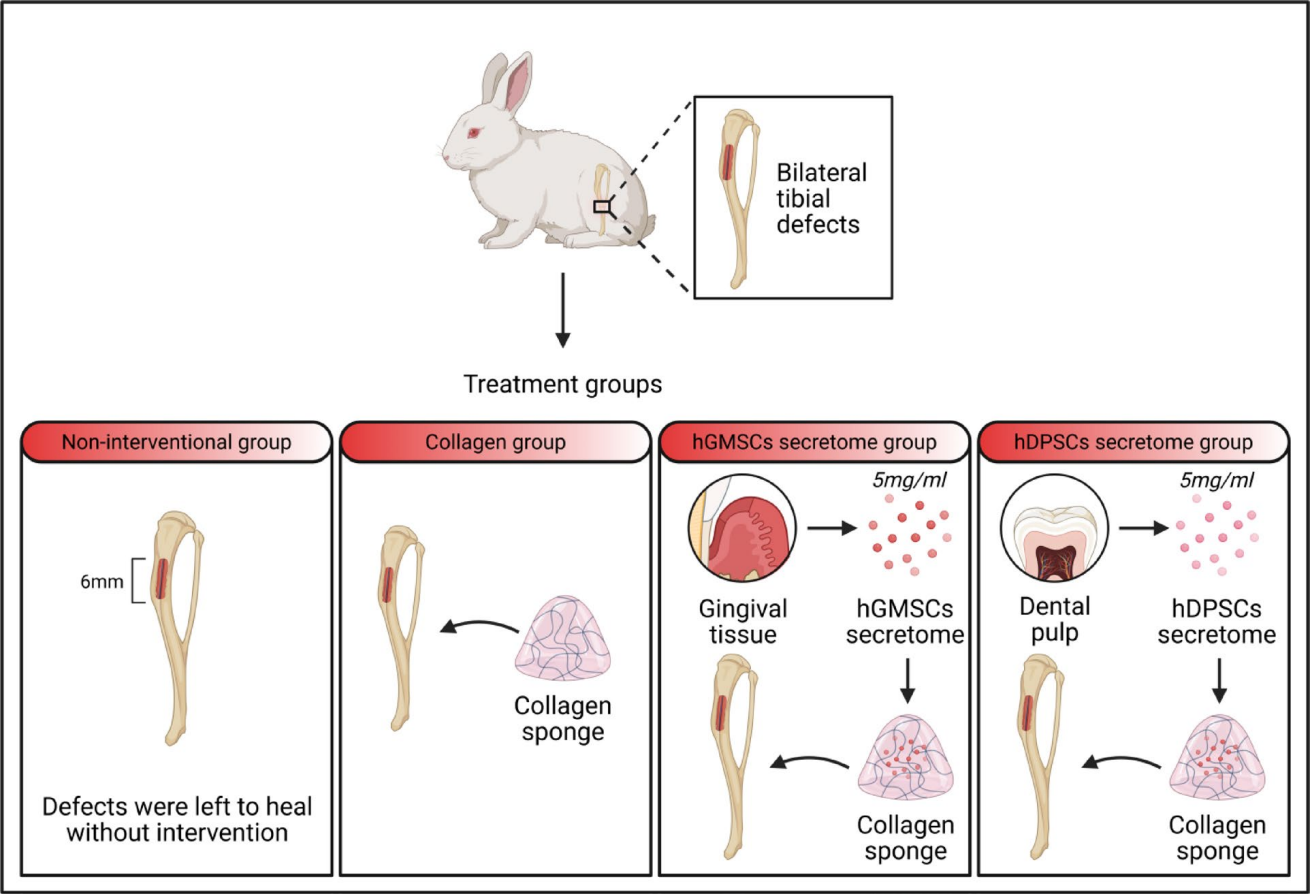
A diagram showing the animal grouping.

The defects were randomly divided into four groups: the non-interventional group, where defects were left to heal without intervention; the collagen group, where defects received only collagen sponges (Collacone^®^: Collagenic hemostat (cone)-botiss biomaterials-Germany); the hGMSCs secretome group, where defects received collagen sponges immersed in 5 µg/µl^21^of previously prepared secretomes; and the hDPSCs secretome group, where defects received collagen sponges immersed in 5 µg/µl^21^of previously prepared secretomes (Fig. 2).

A sterile tweezer was used to adapt the collagen scaffold, and the periosteum and muscles were covered. Resorbable #4.0 catgut was used for suturing, and interrupted #4.0 silk sutures were used to close the skin^22^.

### Post-operative care and fluorochromes injection

After the surgical procedures, the rabbits received an immediate intramuscular injection of systemic antibiotic Amikacin^®^ (Amoun pharmaceutical company) at a dose of 15 mg/kg every 12 h intramuscularly^23^. Additionally, the rabbits received Ketofan^®^ (Amriya Pharm. Ind., Alex., Egypt) (1 mg/kg) per day as an analgesic^24^ for one week. Free movement was allowed for the animals in their cages without external support. The animals were checked throughout the study for signs of limb fractures, illness, and adverse effects. If any were detected, the animals were discarded.

For detecting the mineral apposition rate (MAR), sequential fluorescent labeling of 12 rabbits with a total number of 24 defects divided between the four groups was performed. Subcutaneous injection of 25 mg/kg oxytetracycline (commercially available Oxyvet-Pharma 5%^®^ (Swede- Egypt)) and 20 mg/kg calcein green fresh solution that was prepared just before its injection and was kept in a sealed opaque container (by dissolving 260 mg calcein ^®^ powder (Calcein indicator AR- LOBA CHEMIE PVT. LTD.) and 1012 mg sodium bicarbonate in 500 ml normal saline followed by filtration at 14 and 28 days postoperatively, respectively^25,26^. Half of the rabbits were euthanized after three weeks, and the other half after six weeks^27^. Euthanasia was performed by an overdose of Ketamine/Xylazine mixture (Ketamine chlorhydrate 0.24 ml/100 gm and Xylazine 2% 0.12 ml/100 gm body weight) via the intraperitoneal route. Tibiae were dissected, and soft tissues were removed. Bone specimens, including the defect, were sliced under continuous irrigation.

### Preparation of un-decalcified sections

Areas of the defects with sufficient surrounding sound bone (5 mm above and below) were fixed in a 10% calcium formol solution. After fixation, washing, and dehydration, the specimens were embedded in polymethyl-methacrylate. Transverse sections were prepared by sectioning the bone blocks to 0.3 mm (300 μm) sections using low-speed precision cutter (IsoMet™ 4000, Buehler, North America) (with blade speed = 250 rpm and feed rate = 16.7 mm/min) under water coolant using a diamond wheel blades (AbrasiMet™, Buehler, North America)^28^. Further manual grinding was done using a rotary lathe stone disc fitted on a motor under constant water spray until reaching 0.1 mm (100 μm) thickness. A digital thickness gauge was used to check the required thickness. Then, the specimens were cleaned, dried, and mounted on a slide.

### Examination with an inverted fluorescence microscope and calculation of MAR

Specimens were examined using an inverted fluorescence microscope (Leica - Dmi8 Automated Inverted fluorescence microscope, USA) with an I3 filter cube. The distance between the parallel fluorescent lines (green (in the case of calcein) and orange (in the case of oxytetracycline)) was measured using ImageJ 1.50i (NIH) software, and MAR was calculated by dividing the distance by the time between doses (14 days)^29^.

### Histological and histomorphometry assessment

Fixation of the bone specimens assigned to histological and histomorphometric assessment was carried out with 10% calcium formol solution for 48 h, followed by demineralization via immersion in a solution of 10% EDTA for about four weeks, followed by dehydration, cleared in xylene, embedded in paraffin. The paraffin blocks were then sliced into 5–6 μm-thick sections that were then stained by either Hematoxylin and Eosin (H&E) stain or Masson’s trichrome stain (MT) (H&E: ab245880, MT: ab150686; Abcam, Egypt Distributor; GeneTech Company), using standard procedures for histological analysis. Bone samples were examined using a light microscope coupled with a digital camera (Leica DM300 Microsystems, Inc., Switzerland). The area percentage of newly formed bone from H&E-stained sections was measured while MT-stained sections were used to measure the area percentage of mature bone, immature bone as well as bone marrow spaces. The histomorphometric data were obtained using image analysis software Image J using an objective lens of magnification x20 for H&E MT non-overlapping fields.

### Gene expression detection for osteocalcin using qRT-PCR

RNAs were isolated using a Qiagen tissue extraction kit (Qiagen, USA). Total RNA (1 µg) was subjected to qRT-PCR was performed using an ABI 7600 Fast Thermocycler with iScriptTM One-Step RT-PCR Kit with SYBR^®^ Green (BioWhittaker Molecular Application, Rockland, ME, USA). Both cDNA synthesis and PCR amplification were carried out in the same tube. The expression of the osteocalcin gene was normalized in relation to the mean critical threshold (CT) values of the mRNA for glyceraldehyde 3-phosphate dehydrogenase (GAPDH) as an internal non-interventional by the ΔΔCt technique. Primers for osteocalcin and GAPDH are listed in Table 1.

**Table 1:** Showing the primers used.

Osteocalcin	
Forward primer	5’-CGCTACCTGTATCAATGGCTGG-3’
Reverse primer	5’-CTCCTGAAAGCCGATGTGGTCA-3’
GAPDH	
Forward primer	5’-CATCACTGCCACCCAGAAGACTG-3’
Reverse primer	5’-ATGCCAGTGAGCTTCCCGTTCAG-3’

### Statistical analysis

Data was analyzed using IBM SPSS advanced statistics (Statistical Package for Social Sciences), version 18 (SPSS Inc., Chicago, IL). Data was normally distributed and was described as mean and standard deviation (SD). Data regarding RUNX, osterix, and ALP levels were analyzed using an independent Student’s T-test. Two-way ANOVA was used to assess the effect of different interventions on area percentage of the newly formed bone, area percentage of mature bone, area percentage of immature bone, bone marrow area percentage as well as osteocalcin gene expression in different periods, while One-Way ANOVA was used to analyze MAR data. Tukey’s post hoc test followed this in the case of statistically significant ANOVA results. The value of *p* < 0.05 was considered statistically significant.

## Results

### ELISA results (RUNX, Osterix and ALP levels)

**Fig. 3:**
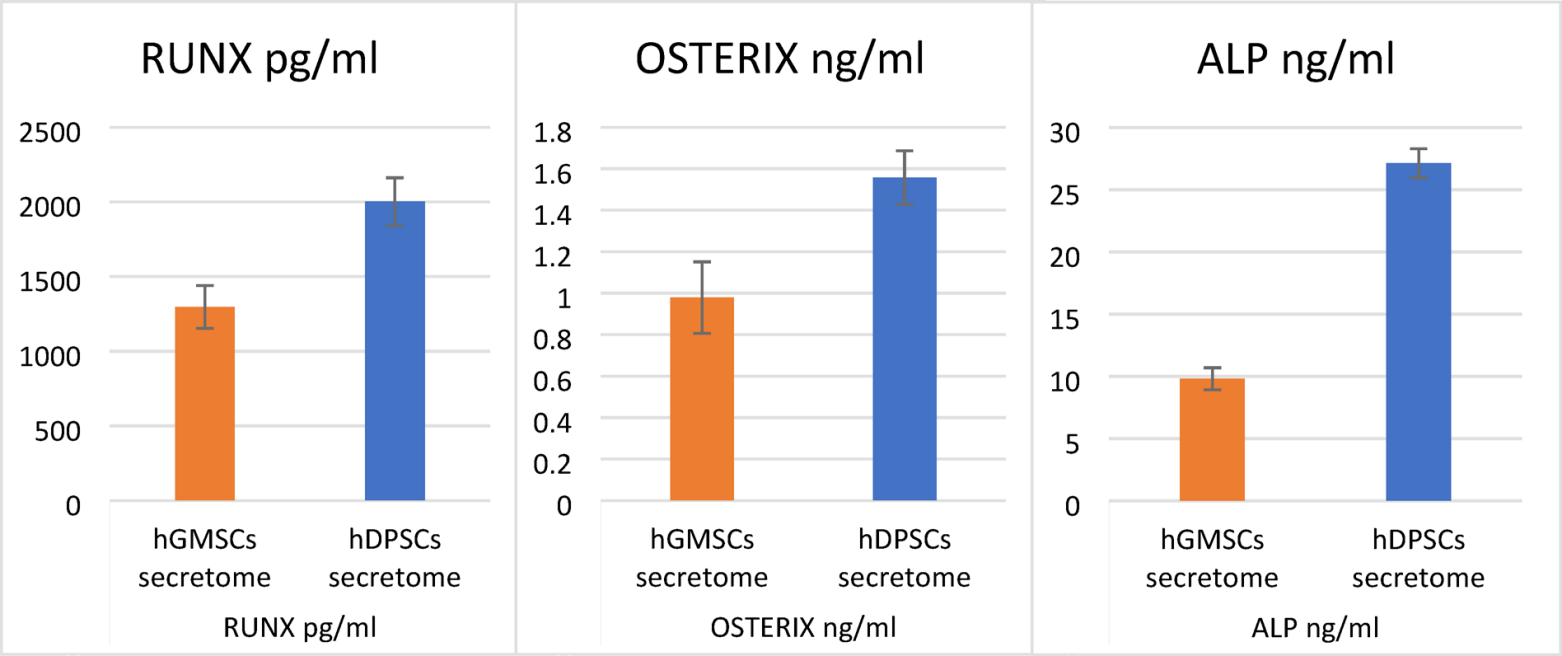
bar chart showing mean values for RUNX, OSTERIX and ALP levels with SD error bars for both gingiva and pulp groups.

A statistically significant higher mean levels of RUNX were observed in the hDPSCs secretome (2002.7 ± 159.0) as compared to the hGMSCs secretome (1296.3 ± 143.0) (*p* < 0.05). Similarly, a significant increase in osterix levels were detectable in the hDPSCs secretome (1.5571 ± 0.1293) as compared to the hGMSCs secretome (0.9786 ± 0.1722) (*p* < 0.05). A significant increase in ALP levels were also observed in the hDPSCs secretome (27.126 ± 1.161) as compared to the hGMSCs secretome (9.813 ± 0.884) (*p* < 0.05) (Fig. 3).

### Mineral apposition rate (MAR)

**Fig. 4:**
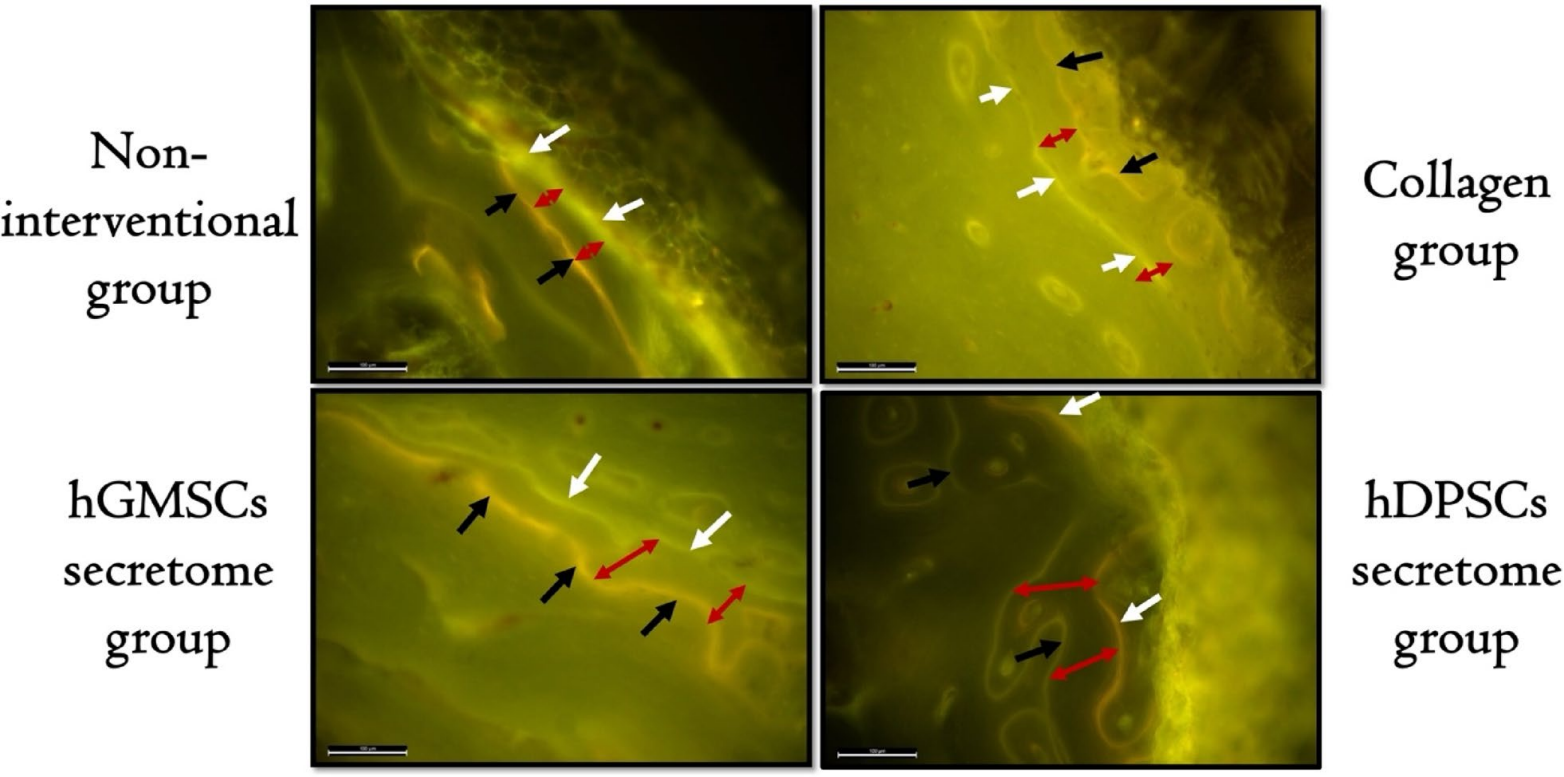
Fluorescence microscopy images showing structural features of the newly formed bone at the induced defects. The black arrows represent orange fluorescent lines indicating the presence of oxytetracycline, the white arrows represent green fluorescent lines indicating the presence of calcein, and the red arrows represent the distance between successive fluorescent lines (LEICA-DMi8- Automated Inverted Fluorescence Microscope, Orig. Mag.200).

The newly formed bone was identified through distinct, well-defined fluorescent markings. Specifically, orange fluorescent lines indicate the presence of oxytetracycline, administered at week 2, while green lines represent calcein, given at week 4 (Fig. 4).

The highest mean MAR was recorded in the hDPSCs secretome group, followed by the hGMSCs secretome group, with a statistically significant difference between groups (*p* < 0.05). One-way ANOVA followed by Tukey’s pair wise comparison revealed a statistically significant higher MAR in the hDPSCs secretome group as compared to the hGMSCs secretome (*p* = 0.018), collagen (*p* < 0.05) and non-interventional groups (*p* < 0.05), also significantly higher value was recorded in hGMSCs group as compared to collagen (*p* < 0.05) and non-interventional groups (*p* < 0.05) while the difference between collagen and non-interventional groups was not statistically significant (Table 2).

**Table 2:** Descriptive statistics and comparison between groups for MAR (one way ANOVA).

Parameter	Group	Mean	Std. Error	One way ANOVA *P* value
MAR	Non-interventional	2.999 ± 1.048 ^C^	0.302	0.000*
	Collagen	3.925 ± 0.819 ^C^	0.236	
	hDPSCs secretome	10.034 ± 0.757 ^A^	0.309	
	hGMSCs secretome	7.780 ± 2.316 ^B^	0.946	

Significance level *P* < 0.05.

*significant means with different superscript letters are significantly different.

### Histopathological and histomorphometric assessment

#### Hematoxylin and Eosin stain

**Fig. 5:**
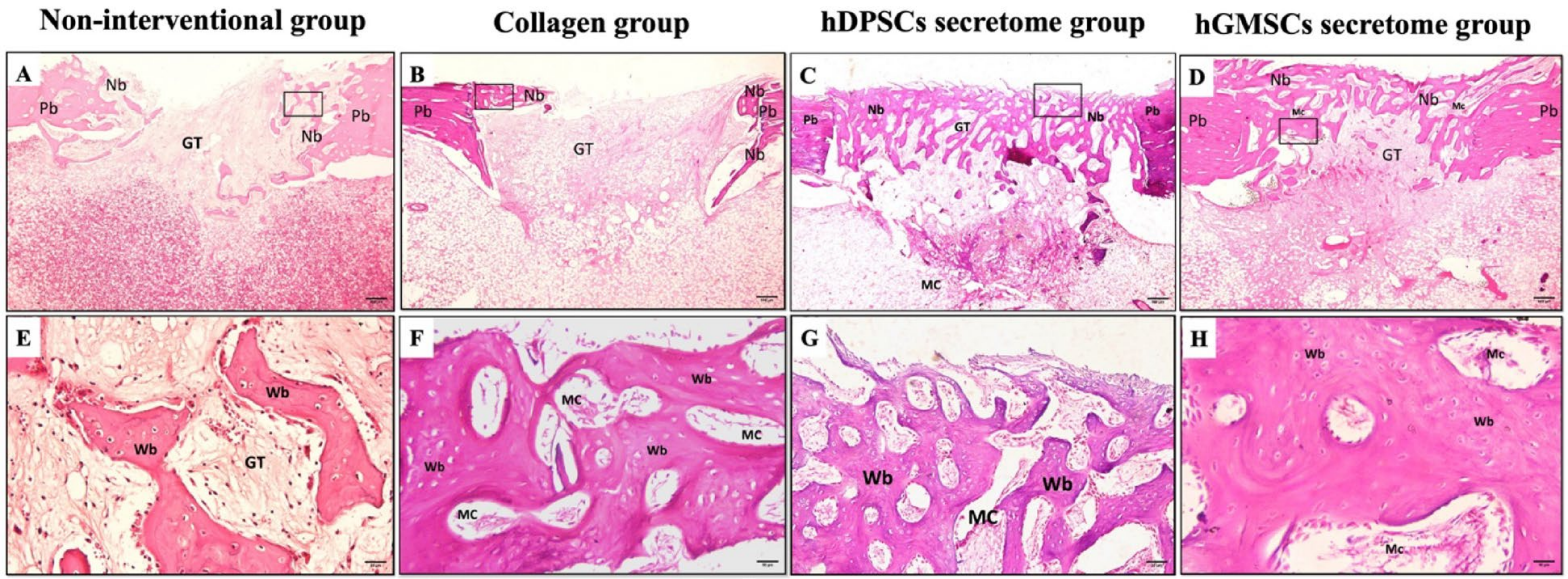
Histological photomicrographs showing defects after 3 weeks in different studied groups; non-interventional group (Panels **A** and **E**), Panel A shows the overall structure of the defect site that is filled with GT with very thin Nb radiating from Pb, Panel E focuses on Nb that of Wb in nature. Collagen group (Panels **B** and **F**), Panel **B** shows the defect filled with GT with very thin Nb radiating from Pb, Panel F focuses on Nb which is Wb in nature enclosing Mc. hDPSCs’ secretome group (Panels **C** and **G**), and hGMSCs’ secretome group (Panels **D** and **H**), Panels **C** and **D** show the defect obliterated with thick Nb, Panels **G** and **H **show thick Nb which is Wb in nature enclosing Mc. (GT: Granulation tissue, Pb: Parent bone, Nb: Newly formed bone, Wb: Woven bone, MC: Bone marrow cells). (Hx & E stain, panels **A**, **B**, **C** & **D**: Orig. Mag. 40 and panels: **E**, **F**, **G** & **H**: Orig. Mag. 400).

Histological interpretation at three weeks revealed unhealed defect areas in non-interventional as well as collagen scaffold groups, while both treatment groups showed bridging of the defect area joining both defect edges together by newly formed woven bone trabeculae (Fig. 5). Statistical analysis of histomorphometry results detected the highest mean bone area percent in hDPSCs secretome group while the lowest value was detected in the non-interventional group with statistically significant difference between groups (*p* < 0.05). Pair wise comparison showed a significantly higher mean area percentage in hDPSCs secretome group as compared to hGMSCs secretome group (*P* = 0.001), collagen (*p* < 0.05) and non-interventional groups (*p* < 0.05). A significantly higher mean value was also recorded in hGMSCs secretome group as compared to collagen (*p* < 0.05) and non-interventional (*p* < 0.05) groups. The difference between collagen and non-interventional groups was not statistically significant (Table 3).

**Fig. 6:**
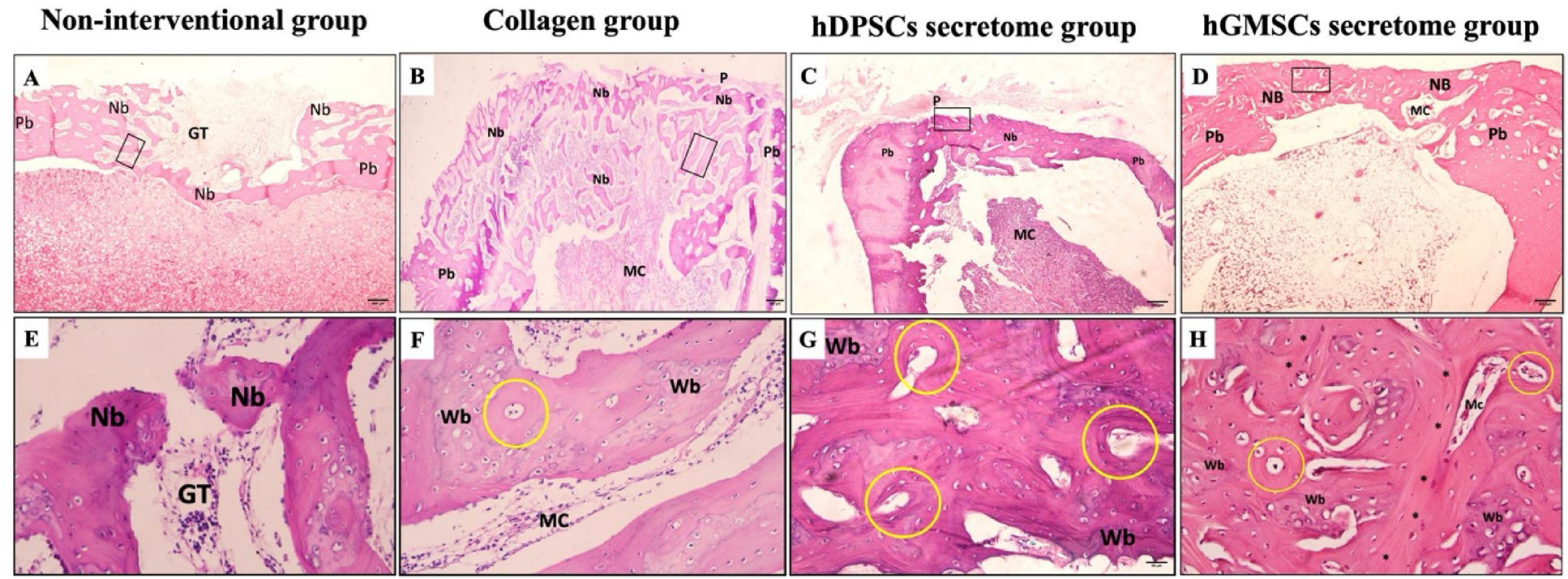
Histological photomicrographs showing defects after 6 weeks in different studied groups; non-interventional group (Panels **A** and **E**), Panel A shows the overall structure of the defect site that is filled with GT with very thin Nb radiating from Pb, Panel E focuses on Nb that of Wb in nature. Collagen group (Panels **B** and **F**), Panel B shows the defect filled with GT with very thin Nb radiating bridging Pb and covered with P, Panel F focuses on Nb which is Wb in nature enclosing Mc. hDPSCs’ secretome group (Panels **C** and **G**), and hGMSCs’ secretome group (Panels **D** and **H**), Panels **C** and **D** show the defect obliterated with thick Nb covered with P, Panels G and H show thick Nb which is Wb in nature enclosing Mc. Some Haversian systems could be detected (yellow circles) (GT: Granulation tissue, Pb: Parent bone, P: Periosteum, Nb: Newly formed bone, Wb: Woven bone, MC: Bone marrow cells). (Hx & E stain, panels **A**, **B**, **C** & **D**: Orig. Mag. 40 and panels: **E**,** F**, **G** & **H**: Orig. Mag. 400).

Regarding six weeks, histological interpretation showed thin bone rim at the base of the defect in the non-interventional group, while the defect in the collagen scaffold group was filled with thin newly formed bone trabeculae. Indeed, the treatment groups showed the defect area entirely filled with newly formed bone that showed more organized patterns at some areas in the form of osteons (Fig. 6). Statistical analysis of histomorphometry analysis showed the highest mean area percentage in hDPSCs secretome group, while the lowest value was observed in non-interventional group with statistically significant difference between groups (*p* < 0.05). Pair wise comparison showed a significantly higher mean area percentage in hDPSCs secretome group as compared to collagen (*p* < 0.05) and non-interventional (*p* < 0.05) groups. A significantly higher mean value was detected in hGMSCs secretome group as compared to collagen (*p* < 0.05) and non-interventional (*p* < 0.05) groups, also significantly higher mean value was detected in collagen compared to non-interventional (*p* = 0.012) group. While the difference between hGMSCs secretome and hDPSCs secretome groups was not statistically significant (Table 3).

A statistically significant increase in mean area percentage was detected within the six weeks’ time as compared to the three weeks’ time in non-interventional group (*p* < 0.05), hGMSCs secretome group (*p* < 0.05), hDPSCs secretome group (*p* < 0.05) and scaffold group (*p* < 0.05) (Table 3).

**Table 3:** Descriptive statistics and comparison between groups for bone area percentage (Two- ways ANOVA test).

Parameter	Duration	Group	Mean	Std. Error	2 ways ANOVA *P* value
Bone area percentage (%)	3 weeks	Non-interventional	18.135 ± 3.154 ^F^	0.843	0.000*
		Collagen	21.66 ± 4.52 ^F^	1.21	
		hDPSCs secretome	57.82 ± 5.11 ^B^	1.93	
		hGMSCs secretome	46.36 ± 5.22 ^C^	1.97	
	6 weeks	Non-interventional	27.84 ± 5.22 ^E^	1.40	
		Collagen	34.72 ± 7.81 ^D^	2.09	
		hDPSCs secretome	85.430 ± 0.680 ^A^	0.257	
		hGMSCs secretome	83.856 ± 2.490 ^A^	0.941	

Significance level *P* < 0.05.

*significant means with different superscript letters are significantly different.

#### Masson’s trichrome stain

**Fig. 7:**
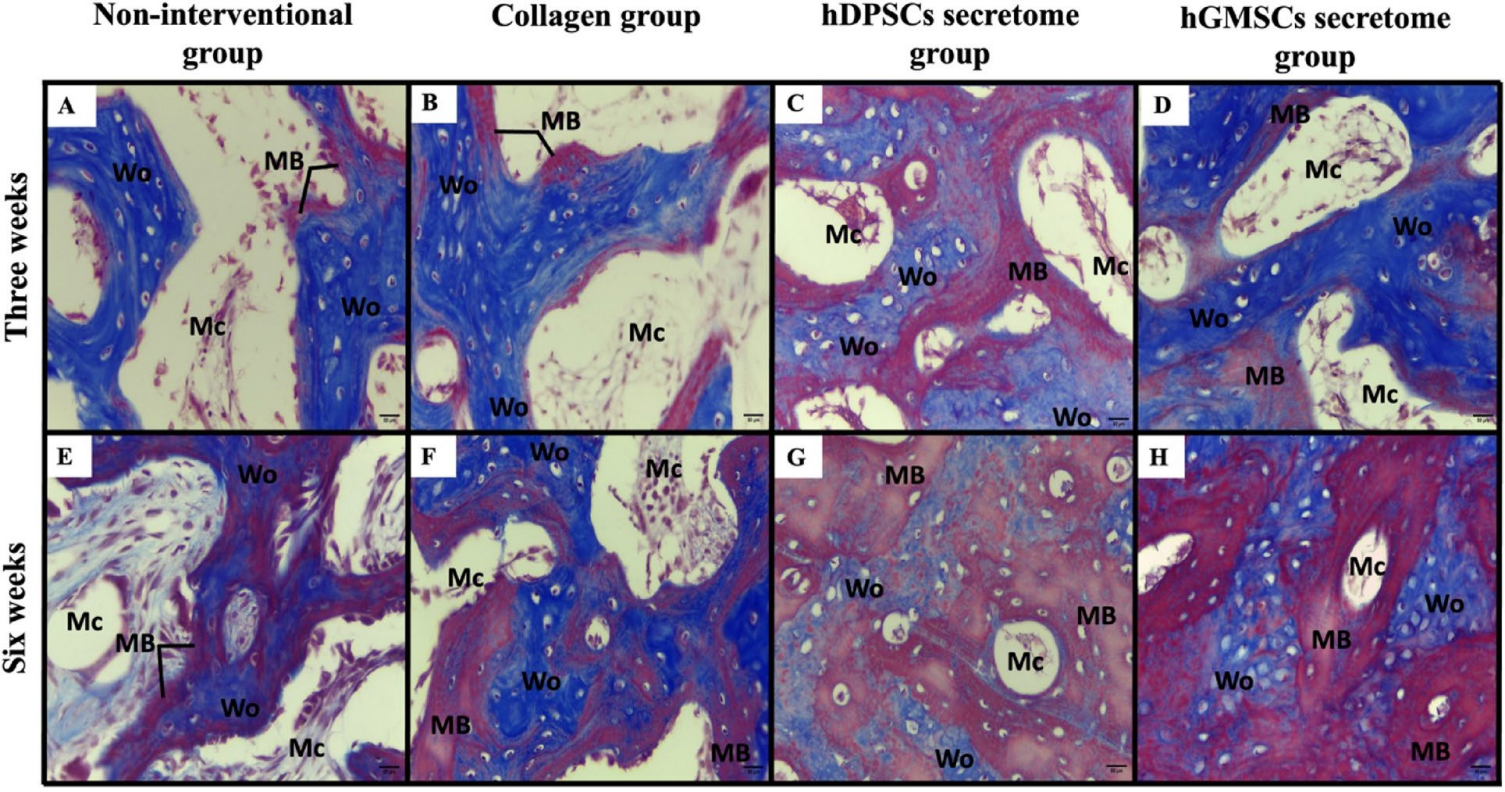
Histological photomicrographs of Masson trichrome stained defects after 3 weeks (Panels **A** to **D**) & after 6 weeks (Panels **E** to **H**). Non-interventional group (Panels **A** & **E**). Collagen group (Panels **B** & **F**), hDPSCs secretome group (Panels **C** & **G**), and hGMSCs secretome (Panels **D** & **H**). (Mb: Newly formed mature bone, Wb: Woven bone, MC: Bone marrow spaces). (MT stain, Orig. Mag. 400).

MT-stained sections revealed various grades of collagen maturation the newly formed bone trabeculae, displayed new collagen fibers formation indicated by a blue color representing an immature woven bone with very thin areas of lamellar bone represented by a red color indicating delayed bone repair (Fig. 7).

#### Mature bone

Statistical analysis of histomorphometry results of 3 weeks’ and 6 weeks’ Masson Trichrome stained sections detected the highest mean mature bone area percentage in hDPSCs secretome group followed by hGMSCs secretome group, while the lowest value was detected in the non-interventional group with statistically significant difference between groups (*p* = 0.013). Pair wise comparison of 3 weeks groups showed a significantly higher mean area percentage in hDPSCs secretome group as compared to collagen group (*p* < 0.05) and non-interventional group (*p* < 0.05). A significantly higher mean value was also recorded in hGMSCs secretome group as compared to collagen group (*p* < 0.05) and non-interventional group (*p* < 0.05), also in collagen group as compared to non-interventional group (*P* = 0.008). The difference between hDPSCs secretome group and hGMSCs secretome group was not statistically significant (Table 4).

Regarding the 6 weeks group, a significantly higher mean mature bone% was detected in hDPSCs secretome group as compared to collagen group (*p* < 0.05) and non-interventional group (*p* < 0.05), significantly higher mean value was also detected in hGMSCs secretome group as compared to collagen (*p* < 0.05) and non-interventional (*p* < 0.05) groups, while no other significant relations were observed (Table 4).

A statistically significant increase in mean mature bone area percentage was detected within the six weeks’ time as compared to the three weeks’ time in non-interventional group (*p* < 0.05), collagen group (*p* < 0.05), hDPSCs secretome group (*p* < 0.05) and hGMSCs secretome group (*p* < 0.05) (Table 4).

#### Immature bone

The highest mean immature bone area percentage in 3 weeks’ and 6 weeks’ groups was observed in hDPSCs secretome group followed by hGMSCs secretome group, while the lowest value was detected in the non-interventional group with statistically significant difference between groups (*p* < 0.05). Pair wise comparison of 3 weeks group showed a significantly higher mean area percentage in hDPSCs secretome group as compared to non-interventional group (*p* < 0.05) and in hGMSCs secretome group as compared to non-interventional group (*p* < 0.05), also in collagen group as compared to non-interventional group (*P* = 0.001). Other pair wise comparisons were not statistically significant (Table 4).

Pair wise comparisons of the 6 weeks’ groups revealed a significantly higher mean immature bone% in collagen group as compared to non-interventional group (*p* = 0.039), while no other significant relations were observed (Table 4).

A statistically significant decrease in mean immature bone area percentage was detected with time in hGMSCs secretome group (*p* < 0.05) and in hDPSCs secretome group (*p* < 0.05) (Table 4).

#### Bone marrow spaces

The lowest mean bone marrow spaces area percentage within the 3 weeks’ group was detectable in hGMSCs secretome group, and in the hDPSCs secretome group within the 6 weeks’ group, while the highest value for both time periods was detected in the non-interventional group with statistically significant difference between groups (*p* = 0.005). Within the 3 weeks groups, pair wise comparison showed a significantly lower mean bone marrow area percentage in hGMSCs secretome group as compared to non-interventional group (*p* < 0.05), and in hDPSCs secretome group as compared to non-interventional group (*p* < 0.05) also in collagen group as compared to non-interventional group (*P* = 0.001). Other pair wise comparisons were not statistically significant (Table 4).

Regarding the 6 weeks groups, a significantly lower bone marrow spaces area percent was detected in hDPSCs secretome group as compared to hGMSCs secretome group (*p* = 0.007), Non-interventional group (*p* < 0.05) and collagen group (*p* < 0.05), also in hGMSCs secretome group as compared to non-interventional group (*p* < 0.05) and collagen group (*p* < 0.05), and in collagen group as compared to non-interventional group (*p* < 0.05), while the difference between hGMSCs secretome group and collagen group was not statistically significant (Table 4).

A statistically significant decrease in bone marrow spaces area percentage was detected within the six weeks’ time as compared to the three weeks’ time in in hDPSCs secretome group (*p* < 0.05) (Table 4).

**Table 4:** Descriptive statistics and comparison between groups for histomorphometry of Masson trichrome stained sections (Two- ways ANOVA test).

Parameter	Duration	Group	Mean	Std. Error	2 ways ANOVA *P* value
Mature bone area percentage (%)	3 weeks	Non-interventional	6.58 ± 3.18 ^**E**^	1.30	0.013*
		Collagen	14.01 ± 4.06 ^**D**^	1.66	
		hDPSCs secretome	28.193 ± 1.179 ^**B**^	0.481	
		hGMSCs secretome	26.71 ± 3.92 ^**B**^	1.60	
	6 weeks	Non-interventional	19.560 ± 2.118 ^**C**,**D**^	0.865	
		Collagen	23.41 ± 3.10 ^**B**,**C**^	1.27	
		hDPSCs secretome	45.07 ± 3.94 ^**A**^	1.61	
		hGMSCs secretome	44.55 ± 3.88 ^**A**^	1.59	
Immature bone area percentage (%)	3 weeks	Non-interventional	24.54 ± 4.07 ^**C**,**D**^	1.66	0.000*
		Collagen	36.02 ± 3.40 ^**A**,**B**^	1.39	
		hDPSCs secretome	42.48 ± 4.50 ^**A**^	1.84	
		hGMSCs secretome	42.28 ± 2.86 ^**A**^	1.17	
	6 weeks	Non-interventional	22.24 ± 5.44 ^**D**^	2.22	
		Collagen	30.11 ± 2.57 ^**B**,**C**^	1.05	
		hDPSCs secretome	29.43 ± 3.64 ^**B**,**C**,**D**^	1.49	
		hGMSCs secretome	25.52 ± 5.54 ^**C**,**D**^	2.26	
Bone marrow spaces area percentage (%)	3 weeks	Non-interventional	70.58 ± 4.91 ^**A**^	2.00	0.005*
		Collagen	51.81 ± 6.53 ^**B**^	2.67	
		hDPSCs secretome	47.53 ± 4.28 ^**B**,**C**^	1.75	
		hGMSCs secretome	46.97 ± 7.62 ^**B**,**C**^	3.11	
	6 weeks	Non-interventional	65.15 ± 6.05 ^**A**^	2.47	
		Collagen	41.94 ± 7.09 ^**B**,**C**^	2.89	
		hDPSCs secretome	25.43 ± 3.79 ^**D**^	1.55	
		hGMSCs secretome	38.45 ± 4.19 ^**C**^	1.71	

Significance level *P* < 0.05.

*significant means with different superscript letters are significantly different.

### Detection of osteocalcin gene expression by qRT-PCR

**Fig. 8:**
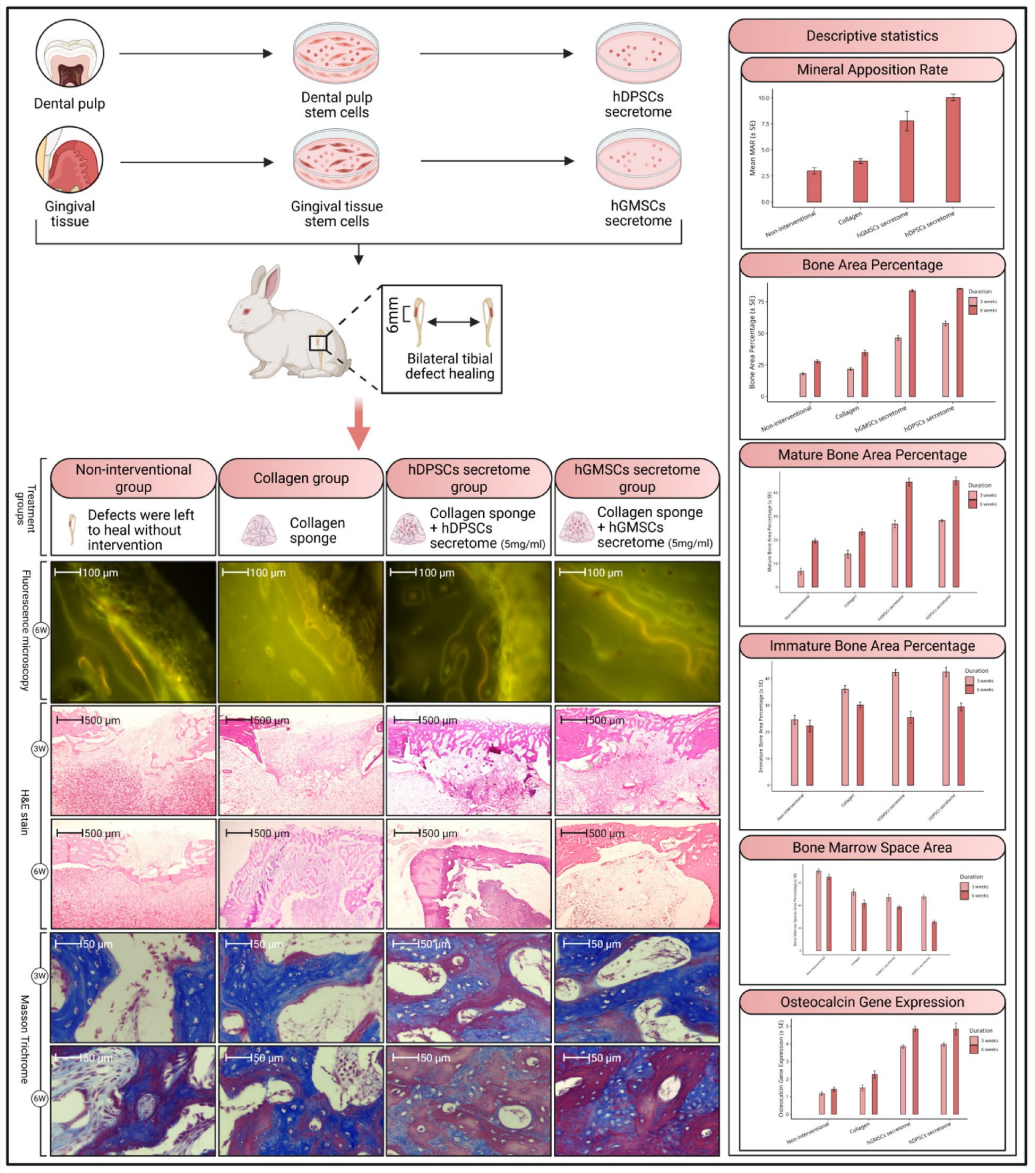
A diagram showing a summary of the methodology and results.

2 ways ANOVA followed by post hoc test within both the three weeks’ time and the six weeks’ time, revealed a significantly higher gene expression in hDPSCs secretome group as compared to collagen group (*p* < 0.05) and non-interventional (*p* < 0.05) groups. A significantly higher gene expression was also recorded in hGMSCs secretome group as compared to collagen group (*p* < 0.05) and non-interventional (*p* < 0.05) groups, while the difference between hGMSCs secretome and hDPSCs secretome groups was not significant in both time periods (Table 5). A statistically significant increase in osteocalcin gene expression was detected within the six weeks’ time as compared to the three weeks’ time in non-interventional group (*p* = 0.005), hGMSCs secretome group (*p* < 0.05), hDPSCs secretome group (*p* < 0.05) and collagen group (*p* < 0.05) (Table 4). The methodology and results are summarized in Fig. 8.

**Table 5:** Descriptive statistics and comparison between groups for osteocalcin gene expression (2 ways ANOVA test).

Parameter	Duration	Group	Mean	Std. Error	2 ways ANOVA *P* value
Osteocalcin gene expression	3 weeks	Non-interventional	1.1767 ± 0.0975 ^E^	0.026	0.000*
		Collagen	1.5017 ± 0.1633 ^D^	0.044	
		hDPSCs secretome	3.9649 ± 0.0983 ^B^	0.037	
		hGMSCs secretome	3.8481 ± 0.0960 ^B^	0.036	
	6 weeks	Non-interventional	1.4233 ± 0.1234 ^D^	0.033	
		Collagen	2.2660 ± 0.2031 ^C^	0.054	
		hDPSCs secretome	4.836 ± 0.336 ^A^	0.127	
		hGMSCs secretome	4.8473 ± 0.1422 ^A^	0.054	

Significance level *P* < 0.05.

*significant means with different superscript letters are significantly different.

## Discussion

Bone defects caused by serious trauma, tumors, inflammation and various other factors have become more common. Bone experiences remodeling, growth and development, displaying exceptional regenerative abilities after injury. A critical size bone defect goes beyond an individual’s regenerative capacity, resulting in poor healing and nonunion^30,31^. Among oral-derived MSCs, GMSCs and DPSCs exhibit significant potential for bone repair and reconstruction in clinical settings. These cells were obtained from gingival tissues and dental pulp, respectively, demonstrating MSCs-like traits and exceptional capabilities in regenerating nerves and bone tissue^32,33^they release numerous active substances such as extracellular vesicles, growth factors, cytokines, and extracellular matrix^34^. These cell-free elements are sourced from different methods and are suitable for multiple formulations, including cell lysates, extracellular vesicles, and conditioned media. Numerous studies have demonstrated that the cell-free elements of MSCs may provide a better therapeutic effect than MSCs in disease treatment^35^.

The current research intended to assess and compare the bone regeneration capabilities of hDPSCs and hGMSCs as a form of cell-free therapy implanted locally in surgically created critical-sized tibial bone defects in rabbits by determining MAR, conducting histomorphometry analysis on newly formed bone areas and measuring osteocalcin gene expression via qRT-PCR.

The safety and efficacy of MSCs secretome in a clinical trial with humans for regenerating alveolar bone and periodontal tissue were previously evaluated using autologous BM-MSCs^36^. No patient exhibited unusual swelling or delayed recovery after the surgery. They determined that MSCs secretome were significantly osteoinductive^36^.

Fluorochrome analysis is an uncomplicated and effective method extensively employed to explore bone formation dynamics alongside histological sections. This technique aids in determining the timing and site of bone formation as well as the rate of formation without requiring the experiment to conclude, by administering fluorescent dyes that adhere to calcium^37^. This method is better than histological sections since histology only offers a final measurement and does not permit dynamic studies. Additionally, the radiological assessment does not enable thorough tissue inspection^25^.

The euthanasia dates selected were 3 and 6 weeks, as stated by Sohn et al., 2010, the initial stages of bone healing are crucial and due to the rapid remodeling phase in rabbits, a healing duration of 2 to 4 weeks would be adequate to assess the healing progress in a rabbit model. As a result, fluorochromes administration (oxytetracycline and calcein green) in this research was performed at 14 and 28 days^38^.

Subcutaneous injection was recommended for small animals to prevent the danger of severe hypocalcemia associated with intravenous administration, or injecting into the intestines, which could result in label absence and potentially fatal peritonitis if intraperitoneal injection was performed^25^. Utilizing MAR to assess the regenerative capacity of a secretome in bone is regarded as a novel research approach, as it has not been previously employed in the existing literature to our knowledge.

Analysis of the un-decalcified bone slices showed the unlabeled older bone with a dark hue and the newly formed bone was marked by oxytetracycline and calcein green. They appeared as thin yellow and green lines, respectively. The distance recorded between the yellow and green lines indicated the quantity of bone created during the time period between the injection of the two fluorochromes. The MAR each day was determined by dividing the label width by the marker interval (14 days). Our findings indicated a statistically significant increase in MAR within the pulp group in comparison to the gingiva (*p* = 0.018), scaffold (*p* < 0.05), and non-interventional groups (*p* < 0.05). Additionally, a notably higher value was observed in the hGMSCs secretome group when compared to the collagen group (*p* < 0.05) and non-interventional groups (*p* < 0.05).

For further confirmation, histological analysis was carried out, at three weeks the non-interventional group indicated unhealed defect regions, while at six weeks, histological interpretation showed a thin bone rim at the base of the defect. These results align with Zhao et al., 2011, who indicated that a critical-sized defect exhibits no more than 10% spontaneous healing throughout an animal’s lifespan^39^.

Similar results were noticed for the collagen group at three weeks, while after six weeks, they were filled with thin newly formed bone trabeculae. These results align with the study by Santos et al., 2015, which indicated the presence of new bone formation exhibiting uniformly distributed osteocytes and being partially surrounded by active osteoblasts after eight weeks^40^. These findings further confirm that a collagen scaffold alone is insufficient to achieve superior outcomes. It is essential to compare the effects of transplanting the scaffold alone with those of transplanting a combination of the secretome and the scaffold. Additionally, ongoing assessment of both the rate and quality of the secretome effect on enhancing the natural healing capacity of bone is required. Whereas both treatment groups exhibited bridging of the defect area connecting the defect edges through newly formed woven bone trabeculae at three weeks, whilst after six weeks, the defect area was entirely occupied by newly formed bone, which demonstrated more structured patterns in certain regions resembling osteons which validated the main role of the secretome in bone regeneration.

Statistical analysis revealed a significantly higher mean area percentage observed in the hGMSCs secretome group compared to the collagen group (*p* < 0.05) and non-interventional (*p* < 0.05) group at both time points. This could be attributed to the hGMSCs secretome potential to enhance the quantity and quality of newly formed bone, as evidenced by both in vitro and in vivo research. They demonstrated higher calcium deposits, assessed through alizarin red staining, when hGMSCs secretome was used along with 3D-polylactic acid scaffold, unlike the other groups. Furthermore, the histological analysis of bone regeneration in calvaria defects revealed improved new bone formation, signified by increased extracellular matrix development and bone contact relative to the other groups at six weeks. It was indicated that hGMSCs secretome promoted the osteogenic process more efficiently than the other groups^41^. Our findings align with the promising results observed with hGMSCs secretome that accelerates the formation of new bone callus by promoting the recruitment of endogenous BM-MSCs^42^.

hDPSCs secretome revealed a statistically significant increase in the mean area percentage of newly formed bone compared with all groups at both time points. This aligns with a study utilizing extracellular vesicles secreted by DPSCs with collagen (DPSC-EVs/COL) in 4.6-mm-diameter critical-sized defects on both sides of the rat calvaria bone, where DPSC-EVs/COL created new bone in the center of the bone defect^43^. These results were consistent with Hiraki et al. (2020), who reported that secretome derived from human exfoliated deciduous teeth increase the amount of newly formed bone as well as the degree of bone maturation as revealed by H&E and MT stain^44^.Furthermore, Imanishi et al. (2021) found that employing extracellular vesicles derived from hDPSCs incorporated into a collagen scaffold in critical-size defects of rat calvaria improved new bone formation in both the center and the edges of the defects, resembling the effect of the cells themselves loaded on the collagen scaffold four weeks post-surgery^43^. In the meantime, there was no statistically significant difference between the secretome of both hGMSCs and hDPSCs six weeks postoperatively.

Osteocalcin levels reflect the ability of osteoblasts to calcify the extracellular matrix. Therefore, this protein is beneficial for evaluating bone turnover and the clinical context of bone loss^45^. At the molecular level, significantly increased osteocalcin gene expression was observed in the hDPSCs secretome group and the hGMSCs secretome group via qRT-PCR when compared to both the collagen (*p* < 0.05) and non-interventional (*p* < 0.05) groups, however, the difference between the hGMSCs secretome and hDPSCs secretome groups was not statistically significant at either time point.

Increased osteocalcin levels could indicate greater new bone development, suggesting a heightened recruitment of osteoblastic cells along with accelerated mineralization and differentiation of osteoclasts. The recruitment of osteoblast cells is essential for osteogenesis^45^.

Our findings showed a statistically significant rise in average levels of RUNX, osterix, and ALP in the hDPSCs secretome compared to the hGMSCs secretome (*p* < 0.05) as identified by ELISA. ALP is one of the most common markers of osteogenesis, representing the level of osteogenic differentiation^46^. The ALP, essential for biomineralization processes, eliminates phosphate groups, resulting in calcification^47,48^. In a similar vein, a previous study indicated that the secretome of hDPSCs elevated osteocalcin and ALP expressions in rat models suffering from periodontitis^49^. Moreover, the secretome of dental MSCs promotes osteogenesis by enhancing the migration and mineralization abilities of stem cells through Transforming growth factor beta 1 (TGF-β1) and boosting the expression of osteogenic genes like Runx2, osteocalcin, and osterix^50,51^. Moreover, increased osteocalcin activity enhances alkaline phosphatase activity, promoting bone formation by osteoblasts^52^; this could elucidate the improved results seen in the hDPSCs secretome group relative to the hGMSCs secretome group and further account for the healing of the critical-sized defects in these groups as the elevated level of osteocalcin integrates with.

Notably, a study conducted in vitro demonstrated that hDPSCs exhibit superior osteogenic potential in comparison to other MSCs, including BM-MSCs and hGMSCs^53^. The main disadvantage of stem cell therapy is the low viability of cells post-implantation. Our findings indicate that the scaffold together with the secretome of both hDPSCs and hGMSCs may persistently release cytokines and growth factors in situ, potentially initiating an osteogenic differentiation process in local MSCs, thereby boosting osteogenic gene expression and mineral deposition^14,54^.

This study demonstrates osteogenic potential of hDPSCs and hGMSCs secretomes in the rabbit model of tibial defect. However, the 6-week study duration limits conclusions on long-term remodeling. Long-term bone stability in subsequent studies would be required to assess. Incomplete characterization of the secretome in part suggests proteomic, transcriptomic approaches to identify core bioactive compounds and related mechanisms. Subsequent studies will need to validate in larger animal models. Overcoming these limitations will help speed up clinical translation of dental MSCs-derived secretomes for bone repair.

Despite promising preclinical outcomes, clinical translation requires addressing key challenges. The cell-free nature of secretome therapy inherently mitigates risks associated with live-cell transplantation as immunogenicity, tumorigenicity, and ethical concerns^13,55,56^ positioning it as a regulatory-friendly alternative. Notably, the first-in-human trial using BM-MSCs secretome for alveolar bone regeneration demonstrated both safety and significant osteoinductive capacity^36^providing a translational roadmap for dental MSC secretomes. Critical next steps include: (1) Standardizing secretome production under GMP conditions to ensure batch consistency; (2) Optimizing scaffold-released pharmacokinetics for human-scale defects using delivery systems like 3D-printed biomaterials^43^; and (3) Validating efficacy in large-animal load-bearing models. Given the accessibility of dental tissues during routine oral procedures, DPSCs/GMSCs secretomes offer a scalable off-the-shelf solution for targeted applications like alveolar ridge preservation and periodontal regeneration^57–59^where collagen scaffolds are already clinically established.

## Conclusion

In conclusion, utilizing the secretome from dental MSCs in therapy seems to be a hopeful strategy in regenerative medicine. Compatibility between the donor and recipient is unnecessary as rejection is not possible. Additionally, it reduces the likelihood of tumor formation and the transmission of infectious illnesses. Cell-free therapy is a cost-effective, efficient, and non-invasive method for gathering cells^60^. DPSCs and GMSCs release biomolecules that may initiate a paracrine effect, influencing cell-to-cell communication in the process of repairing damaged tissues. However, many challenges remain that need to be tackled before the clinical application of cell-free therapy. These obstacles include the lack of standardized methods for producing the MSCs-derived secretome, concerns regarding product stability and storage, as well as critical quality non-interventional measures needed to verify the safety and efficacy of MSCs-derived secretome.

## Data Availability

All data will be available from the corresponding author upon reasonable request.
